# Greenhouse Vertical Cultivation to Improve Production Earliness and Fruit Yield and Quality of Melon

**DOI:** 10.3390/plants15040562

**Published:** 2026-02-11

**Authors:** Alessandro Borrelli, Lucia Santonicola, Elen Jones Evans, Luigi G. Duri, Farzaneh Zamani, Antonio G. Caporale, Ivana Ledenko, Luana Izzo, Roberta Paradiso

**Affiliations:** 1 Department of Agricultural Sciences, University of Naples Federico II, 80055 Naples, Italy; alessandro.borrelli2@unina.it (A.B.); farzaneh.zamani@unina.it (F.Z.); ag.caporale@unina.it (A.G.C.); 2 Bayer Crop Science S.r.l., 04100 Latina, Italy; lucia.santonicola@bayer.com (L.S.); elen.jones-evans@bayer.com (E.J.E.); 3 Department of Agriculture, Food, Natural Resources and Engineering (DAFNE), University of Foggia, 71100 Foggia, Italy; luigi.duri@unifg.it; 4 Department of Pharmacy, University of Naples Federico II, 80131 Napoli, Italy; ivana.ledenko@unina.it (I.L.); luana.izzo@unina.it (L.I.)

**Keywords:** *Cucumis melo* L., phenolic compounds, nutritional traits, mineral composition, genotype × environment interaction, sustainable horticulture

## Abstract

Melon (*Cucumis melo* L.) is a major horticultural crop cultivated in temperate and subtropical regions, with increasing importance due to its nutritional value and consumer demand. Currently, melon is grown both in the open field and greenhouse, which differ significantly in agronomic practices, production cost, environmental impact, and fruit yield. Recently, vertical cultivation in greenhouses has been tested as an alternative to traditional trailing systems. This study aimed to evaluate the performance of four melon hybrids, named 2001, 2003, 2005, and 2008, in two soil-based cultivation systems, the traditional trailing growth habit in the open field, and vertical cultivation in the greenhouse. Results revealed significant differences between cultivation systems in fruit yield and quality. Vertical cultivation resulted in a higher yield compared to the open field (7.8 vs. 6.6 kg m^−2^). Fruits harvested from vertically grown plants showed higher levels of total phenolic compounds, enhancing their nutraceutical value. Among the tested hybrids, 2008 reached the highest yield (8.7 kg m^−2^) along with notable nutritional and nutraceutical properties, while 2001 and 2003 showed a superior quality profile, with higher concentrations of K, P, and phenolic compounds. Overall, hybrid 2008 exhibited the best agronomic response across both cultivation systems, combining higher productivity and fruit quality, and can therefore be considered the most suitable genotype for both open-field and vertical farming conditions. The observed genetic variability among the hybrids underscores the importance of selecting the plant genotype best adapted to the chosen cultivation system.

## 1. Introduction

Melon (*Cucumis melo* L.) is one of the most economically and nutritionally important horticultural crops worldwide, particularly in temperate and subtropical regions [1]. It belongs to the Cucurbitaceae botanical family, and its summer fruit is valued for its sensory attributes. Among the many varieties, cantaloupe and netted melon are the most consumed, although the *flexuosus*, *acidulus*, and *conomon* types are often used when underripe in salads or cooked preparations, while the *cantalupensis*, *reticulatus*, and *inodorus* varieties are typically consumed ripe and fresh due to their high sugar content and aromatic features [2].

The sugar profile is a key determinant of fruit quality in sweet melons [3]. Immature fruits primarily contain glucose and fructose, while sucrose accumulation in the mesocarp occurs during the final maturation stage, and continues until harvest or natural abscission [4,5]. Due to the absence of starch reserves in the fruit mesocarp, the sugar content does not increase after harvest. Thus, optimal harvest timing is critical since early harvesting can result in reduced sweetness, while delayed harvesting may compromise the fruit shelf life. Although the relative parts of these soluble sugars may justify some taste variation at an equal total sugar content [3,5,6], the content of total soluble solids (TSS) is a dependable quality marker [7] and can be quickly quantified by extracting pulp juice onto a refractometer.

Melon pulp is a significant source of vitamins and dietary fiber, with a low fat and caloric content [8]. According to traditional Chinese medicine, it was used for pain relief and as a diuretic [9]. It is also rich in phytochemicals, such as polyphenols and β-carotene, recognized for their strong antioxidant properties [8]. In this respect, polyphenols contribute to body protection against oxidative stress and have documented anti-inflammatory, antitumor, and cardiovascular effects [10].

Melon is particularly rich in vitamin A, providing approximately 112% of the recommended daily allowance (RDA) per 100 g. This nutrient supports vision, skin, and mucous membrane health and may reduce the risk of respiratory cancers. Additionally, a melon portion of 100 g offers about 61% of the RDA for vitamin C, which bolsters immune function and limits oxidative damage [11].

Flavonoids, including β-carotene, lutein, zeaxanthin, and cryptoxanthin, further enhance protection against serious pathologies including cancers [12]. Melon is also a good source of potassium, a key electrolyte for regulating blood pressure and cardiovascular health, providing about 270 mg per 100 g [13].

Due to its sensorial attributes and nutritional profile, melon is a globally popular fruit [14]. In 2022, China led the global annual production (14.20 million tons), followed by Turkey (1.59 million tons), India (1.50 million tons), and Kazakhstan (1.21 million tons), while in Europe, Spain ranked first (664,000 tons), and Italy followed (593,000 tons) [15].

In Italy, melon cultivation is widespread in both the open field and greenhouse with a total area of 22,888 ha, including 20,520 ha in the open field and 2368 ha under greenhouses and tunnels, corresponding to 13.7% of the total production in protected cultivation [16].

Melon production, however, faces considerable issues in the open field [17] since outdoor systems are vulnerable to biotic and abiotic stressors, seasonal growth restrictions, and unpredictable weather conditions [18]. Furthermore, open-field crops often require more intensive phytopathological treatments and are concentrated in regional growing period, particularly in cooler or more variable climatic areas. Conversely, protected cultivation allows better control over climate conditions (temperature, humidity), enhancing plant performance in terms of early production and yield, while decreasing the disease frequency and climatic stress [19].

Vertical plant training in greenhouses is a novel approach that allows higher planting densities and optimizes space utilization and resource use efficiency [20]. Training melon plants vertically on trellises or supports enhances air circulation, water distribution, and nutrient management. This practice is applicable for high-value horticultural crops like melon, where earliness, uniformity, and postharvest quality are fundamental market requirements [21].

Despite these promising advantages, comparative studies evaluating the agronomic and qualitative performance of melon under greenhouse vertical training systems are limited. Particularly, data comparing ungrafted plants grown in vertical greenhouse systems compared to traditional open-field horizontal growth methods are not available in the literature, and the influence of these systems on harvest earliness, yield potential, and fruit nutritional and nutraceutical composition is not known in the different commercial genotypes.

The aim of this research is to fill this gap by investigating the agronomic and qualitative responses of four ungrafted melon hybrids under two growing systems, open-field cultivation with horizontally sprawling plants and greenhouse vertical cultivation with supported plants. Our study evaluated the production earliness and fruit yield and quality and provided a trade-off of benefits and limits of each system to guide growers toward more efficient and sustainable production strategies.

## 2. Results

### 2.1. Environmental Parameters: Temperature and Relative Humidity

**Figure 1:**
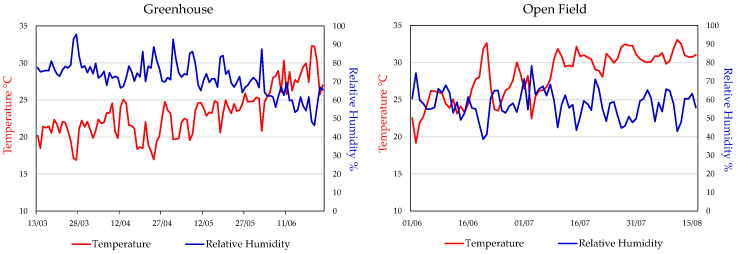
Average daily values of temperature and relative humidity in the two growing environments: greenhouse (13 March–24 June) and open field (1 June–16 August).

Figure 1 shows the temperature and relative humidity values recorded in the greenhouse and open field during the two related cultivation periods. In the greenhouse, data were collected daily during the sunlight period, from 6:00 a.m. to 6:00 p.m. from March 13 to June 24, while in the open field, data were recorded from 7:00 a.m. to 7:00 p.m. from June 01 to August 16. In the greenhouse, the average temperature ranged from 15 to 25 °C for most of the period, gradually increasing to 30 °C from May to June. The relative humidity fluctuated between 70 and 90%, showing a gradual decline towards the end of the cultivation period. In the open field, the average temperatures ranged from 20 to 30 °C, with peaks above 30 °C in July and August. The relative humidity ranged between 40% and 80%, with frequent fluctuations caused by wind, particularly in the afternoon.

### 2.2. Plant Growth

Statistical analysis showed a relevant interaction between the cultivation system (S) and hybrids (H) for the different analyzed parameters except for Soil Plant Analysis Development (SPAD) index, which did not show statistically significant differences.

The results reported in Table 1 show that, overall, the fresh weight, dry weight, and dry matter percentage of the whole plant (aerial part) were significantly higher in trailing plants grown in the open field compared to those trained vertically in the greenhouse. For example, trailing plants in the open field recorded a higher fresh weight per plant (+346.9% compared to vertically grown plants in the greenhouse). The dry matter percentage also showed marked differences, with 21.3% in the open field versus 10.4% under vertical cultivation.

Considering the two cultivation systems separately, the different hybrids showed statistically significant responses for all analyzed parameters.

Under open-field conditions, hybrids 2001, 2005, and 2008 showed the highest SPAD values, while 2003 recorded the lowest. In addition, the fresh weight was slightly higher in 2001 and 2008 compared to 2003 and 2005, whereas the dry weight was significantly higher in hybrid 2008 compared to the others. Finally, the dry matter percentage was higher in hybrid 2008 than in 2005 and 2003, with the lowest value recorded for 2001.

Under vertical farming conditions, hybrid 2005 showed the highest SPAD value, while hybrid 2001 showed the lowest, compared to hybrids 2003 and 2008. The fresh weight was slightly higher in hybrids 2001 and 2008 than in 2003 and 2005, consistent with the trend observed under field conditions. Furthermore, the fresh weight was particularly lower in hybrid 2005 compared to hybrids 2001, 2003, and 2008, which recorded the highest values. Finally, the percentage of dry matter showed a similar trend across all four hybrids.

### 2.3. Fruit Yield, Dry Matter Content, and Refractometric Index

Data analysis revealed that the interaction between the cultivation system (S) and hybrid (H) had highly significant effects on all the growth parameters analyzed, including fresh weight, dry weight, dry matter percentage, and fruit weight (Table 2), with the sole exception of the number of fruits and fruit yield, for which the interaction was not significant.

In open-field conditions, a higher fruit weight, number of fruits, and pulp dry matter content were recorded compared to vertical cultivation. On the other hand, under vertical cultivation, when total production was considered on an annual basis, a slightly higher fruit yield and Brix degrees were observed compared to open-field cultivation. Particularly, in the open field, hybrids 2003 and 2005 showed a higher fresh weight compared to hybrids 2001 and 2008, whereas the number of fruits and fruit yield were higher in hybrid 2008 compared to the others, although not statistically significant. Hybrid 2005 recorded a higher pulp dry matter content and Brix degrees compared to the others.

Conversely, vertical cultivation showed a higher fresh weight for hybrid 2005 compared to hybrids 2001, 2003, and 2008. Hybrids 2001 and 2008 exhibited a higher number of fruits compared to the others, whereas fruit yield was particularly high in hybrid 2008, although not statistically significant. Moreover, hybrid 2005 showed a higher pulp dry matter content and Brix degrees compared to the others.

### 2.4. Multielement Profile of Fruits

The analysis of carbon (C) and macronutrient content in the pulp (Table 3) revealed that the cultivation system significantly influenced the levels of nitrogen (N), phosphorus (P), magnesium (Mg), and sodium (Na), which were higher under greenhouse vertical cultivation compared to conventional open field conditions. For example, the N content in fruits from plants grown in the vertical system reached 18.3 g kg^−1^, compared to 14.6 g kg^−1^ in the open field. Similarly, the P and Na concentrations were higher in vertical cultivation, and a similar trend was noted for Mg.

Significant differences in the pulp composition were also found among hybrids. Hybrid 2003 stood out for its high P content (3.76 g kg^−1^) and shared the highest accumulation of potassium (K), N, calcium (Ca), and Mg with hybrid 2001. Additionally, 2003 showed the lowest Na concentration. Hybrid 2005 was characterized by the highest Ca and the lowest K level. No significant differences in C content were observed among the hybrids, while the P level exhibited a descending gradient across genotypes. The interaction between cultivation system and hybrid (S × H) was statistically significant for several macronutrients (e.g., N, P, and Mg), while not significant for the microelements.

Table 4 presents the concentration in the pulp of key microelements detected, such as iron (Fe), manganese (Mn), boron (B), zinc (Zn), copper (Cu), molybdenum (Mo), and selenium (Se). The cultivation system significantly influenced accumulation, with higher values in fruits from plants grown in the greenhouse compared to those from the open field. In contrast, Cu showed the opposite trend, with higher concentrations recorded in the open field compared to the greenhouse. No significant differences between the two cultivation systems were found for the remaining microelements.

Significant differences were also observed among hybrids for all analyzed microelements. Hybrids 2001 and 2003 showed significantly higher concentrations of Fe, Mn, Zn, and Se, whereas hybrids 2005 and 2008 accumulated lower levels of these elements except for Mo, which was highest in hybrid 2008. Additionally, hybrid 2001 recorded the highest Cu content, while the lowest was measured in hybrid 2008.

Regarding the concentration of macroelements, Na, and C in the exocarp (Table 5), the cultivation system had a highly significant effect on K, P, Mg, and Na, with higher values under vertical greenhouse cultivation. For instance, the P content reached 6.98 g kg^−1^ under vertical cultivation compared to 3.74 g kg^−1^ in the open field. Conversely, Ca was higher under open-field conditions than in the vertical system.

When comparing hybrids, N showed highly significant differences, with hybrids 2008 and 2001 exhibiting the highest values (24.3 g kg^−1^ on average). Significant differences also emerged for K, Mg, and Na, with hybrid 2005 showing the lowest K concentration and the others with similar values and hybrid 2003 reaching the highest Mg content, while the remaining hybrids had comparable values. Differences were less pronounced for Na, with the only significant contrast between hybrid 2005 (highest) and 2001 (lowest).

The interaction between the cultivation system and hybrid (S × H) was not significant for most macroelements, except for P and Mg. Specifically, vertical cultivation resulted in the highest content of P for hybrid 2008 (8.48 g kg^−1^) and Mg for hybrid 2003 (4.77 g kg^−1^).

The analysis presented in Table 6 regarding microelement concentration in the peel shows that the cultivation system significantly influenced most of the measured elements. Among them, Mo stands out in particular: under vertical cultivation, its concentration was nearly 10 times higher than that in open-field conditions (0.695 vs. 0.070 ppm). Vertical cultivation also promoted a higher accumulation of Mn, B, Zn, and Se. In contrast, Cu levels were higher in the open field than in the greenhouse.

Hybrids exhibited differences in microelement accumulation in the peel: hybrid 2008 stood out with the highest Mo content, while the other hybrids had similar values; 2003 had the highest B concentration; 2001 showed significantly higher levels of both Fe and Cu. In absolute terms, Mn was most accumulated in hybrid 2003, although it did differ significantly only from hybrid 2005. A similar trend was observed for Zn, which showed lower levels in hybrids 2005 and 2008 compared to hybrid 2001. Se was significantly lower in the peel of hybrid 2005 compared to the others.

Regarding the interaction between the cultivation system and hybrid (S × H), it was highly significant for Mo, with hybrid 2008 in the greenhouse showing higher values than any other combination. Significant interactions were also found for Mn and B, with hybrid 2003 in the greenhouse recording the highest values. No significant interactions were observed for the other microelements.

### 2.5. Phenolic Profile of Melon Fruits

Quantification of specific polyphenols within the melon sample extracts was performed using UHPLC-Q-Orbitrap HRMS. To ensure both precision and accuracy, calibration curves were prepared in triplicate across eight concentration levels, demonstrating excellent linearity with regression coefficients (R^2^) exceeding 0.990. Untargeted analysis in full-scan HRMS mode enabled the identification of vanillic acid and provided comprehensive data for the retrospective analysis of additional compounds. Structural characterization of untargeted compounds was confirmed through accurate mass measurements, elemental composition assignment, and interpretation of MS/MS spectra.

Quantitative results are presented in Table 7, with average contents expressed in micrograms per gram (μg g^−1^) of extract. These findings provide valuable insights into the polyphenolic composition and concentrations in the extracts derived from melon samples, supporting the reliability and accuracy of the quantification process. The data revealed a significant interaction between the cultivation system and hybrid only for vanillic acid. This pattern appears to be largely driven by vanillic acid itself, which was found at much higher concentrations than other identified phenolic acids and flavonoids.

## 3. Discussion

This study analyzed the productive and qualitative performance of four hybrids of melon grown under two different cultivation systems: the traditional open-field method with trailing plants on the ground and greenhouse vertical cultivation. The primary aim was to evaluate the plant response to these cultivation systems, which differ in both growth environments and plant management practices. Additionally, the study also explored the specific response based on genotype intrinsic features, highlighting the advantages and limitations of each system on the specific genetic material.

Open-field cultivation of melon is particularly suited in subtropical regions, allowing for the extensive use of land to produce high quality produce [22]. However, it is limited by climatic factors such as unfavorable weather conditions and higher susceptibility to phytopathogenic attacks [23]. In contrast, vertical cultivation in protected environments (greenhouse, tunnel) offers several advantages over the open field, including a higher planting density and better space utilization [24], which is particularly beneficial in areas where cultivable land is limited [25]. Experimental results showed more uniform fruit growth and improved quality traits in the open field, such as color, firmness, and sugar content, likely due to better exposure of the canopy to solar radiation [26].

In our experiment, analyzing the differences in the trend of climatic parameters in the two growth environments is essential for interpreting results. In the greenhouse (13 March–24 June), the temperature (T) and humidity (RH) remained relatively stable, with a few peaks of T exceeding 30 °C, preventing climate-related stress and supporting uniform plant development. In the open field (30 May–16 August), greater fluctuations of both T and RH may have negatively affected plant growth and increased crop sensitivity to pathogens. Nevertheless, environmental stress does not always have negative effects, as it can promote the biosynthesis of secondary metabolites, which are useful for increasing the plant’s tolerance, with beneficial effects in human health [27].

The SPAD index, an indirect measure of the chlorophyll content based on leaf greenness and nitrogen content in plant tissue and a useful marker of the plant nutritional status [28], revealed no sign of nutrient deficiency regardless of the cultivation system.

Plants grown in the open field exhibited a significantly higher biomass compared to those in the greenhouse. This difference can be attributed to the lower planting density, which reduced the plant competition for nutrients [29], as well as to the cultivation practice, since plants grown vertically were pruned at the top to be conformed to the system. Besides, possible water and environmental stress in the field likely contributed to the accumulation of soluble sugars and a corresponding increase in the dry matter content [27]. However, in terms of fruit production, greenhouse vertical cultivation outperformed the traditional ground-level growth in the open field (+18.1% in fruit yield per m^2^, on the average of the 4 hybrids). This is mainly due to the more homogeneous climatic conditions, as well as the higher planting density (2.2 vs. 0.5 plants m^−2^ in the open field). In this respect, while the higher density notably reduced the vegetative biomass, it did not affect fruit production, allowing for a greater number of fruits per unit area (m^2^) in the greenhouse [30].

In our experiment, the varietal response was critically important for fruit quality. For hybrids 2001 and 2003, the refractometric index (in Brix degrees), which indicates the total soluble solids content, was higher in fruits from plants in the open field, whereas 2005 exhibited higher values in those from the greenhouse. Hybrid 2008, on the other hand, showed no sensitivity to the growth environment, with a similar refractometric index in both systems. This evidence highlights the need to balance yield and quality based on the available plant genotype and cultivation environment and on specific commercial objectives.

Fruits grown under protected conditions exhibited higher concentrations of N, P, Mg, and Na compared to those from the open field, suggesting that the controlled environment improved nutrient uptake, likely due to the reduced leaching and lower weed competition as well as better fertigation management [30]. Furthermore, fruits from the vertical greenhouse system exhibited higher uptake of Fe, B, Mo, and Cu. Notably, the B content was 46% higher than that in fruits from the open field, confirming better fertilization in the protected system. These findings underscore the significant influence of the growing environment on plant physiological responses and the importance of tailoring cultivation practices to the desired produce features [31].

Among the plant genotypes, hybrid 2005 showed the lowest K content, possibly due to inherent genetic traits or a higher sensitivity to heat stress [32].

The analysis of macro- and microelements in the fruit peel revealed a different pattern compared to the pulp. The Ca and K contents were significantly higher in fruits from the open field. This is likely related to the climacteric ripening process, which is influenced by environmental factors that may stimulate the accumulation of Ca and K in the peel as an adaptive response to enhance the cell wall strength and improve mechanical and osmotic resistance [33].

Micronutrient data showed that fruits produced in the greenhouse exhibited higher levels of all elements except Cu, probably due to the more frequent application of phytosanitary treatments in the open field: for instance, copper-based products (e.g., copper oxychloride, Cu_2_(OH)_3_Cl) were used in the field to prevent and control fungal diseases, such as downy mildew (*Pseudoperonospora cubensis*). However, Cu_2_(OH)_3_Cl leaves residues on plant tissues, especially on the peel, which is often rougher and more retentive [34].

In the hybrid comparison, once again, hybrid 2005 demonstrated a lower capacity to accumulate most micronutrients, likely due to genetic traits limiting the nutrient uptake or translocation within the fruit [35].

Within the broad class of bioactive compounds, polyphenols are of considerable interest due to their protective and preventive effects against chronic diseases, including cardiovascular disorders, cancer, and neurodegenerative conditions in the human body [36]. Although the total polyphenol content in the analyzed melon samples was relatively low, the presence of vanillic acid is noteworthy for its documented antioxidant, anti-inflammatory, and antitumor properties.

The total phenolic compound content in the samples varied significantly, ranging from 18.96 µg g^−1^ to 109.72 µg g^−1^. This variability is consistent with findings by Wang et al. [37], who reported free phenolic content in the pulp of six melon varieties grown in China’s Hainan Province, with values ranging from 193.29 µg g^−1^ to 1830.11 µg g^−1^.

The cultivation system showed a significant effect only on the flavonoid content. Melons grown on vertically trained plants in the greenhouse had a significantly higher flavonoid concentration (11.37 µg g^−1^) compared to those cultivated in the open field (0.37 µg g^−1^). This result aligns with findings by Mallek-Ayadi et al. [13], who suggested that the growing environment may differentially influence various classes of phenolic compounds. Therefore, it can be hypothesized that greenhouse cultivation using vertical architecture may promote greater flavonoid biosynthesis.

The analyzed hybrids displayed significant differences across nearly all parameters, indicating a genetic aptitude for synthesizing specific phenolic acids [21]. Vanillic acid may enhance the nutraceutical quality of the fruit [38,39], making melon fruits not only pleasant to be consumed but also a functional food with recognized health promoting properties [40]. In our samples, vanillic acid emerged as one of the most abundant compounds, reaching a maximum level of 99.30 µg g^−1^. This value exceeds those reported by Mallek-Ayadi et al. [13], who found concentrations of 7.24 mg 100 g^−1^ in the pulp of the ‘*Maazoun*’ melon cultivar. Conversely, Wang et al. [37] detected free vanillic acid in only one sample, at a concentration of 0.38 µg g^−1^. In their study, the predominant compound was a vanillin derivative, ethyl vanillin, with concentrations up to 123.90 µg g^−1^. Rodríguez-Pérez et al. [41] also identified hexoxide derivatives of vanillic acid, confirming its occurrence in multiple forms in melons, including the ‘*Cantaloupe*’ variety. In our study, hybrids 2001 and 2008 consistently exhibited a high vanillic acid content in both open-field and greenhouse systems. Hybrid 2001 stood out as having the highest total phenolic content. In addition to its inherent genetic traits, this elevated production may be linked to a cultivar-specific response to environmental stress (such as excess ultraviolet radiation), which may have stimulated secondary metabolism pathways to synthesize these compounds as a means of adaptation or protection against oxidative stress [42].

## 4. Materials and Methods

### 4.1. Plant Material and Crop Management

The experiment was conducted during the spring–summer season of 2024 at the Bayer Research Centre in Latina (Italy). We compared two cultivation systems, vertical greenhouse cultivation and traditional crawling open-field cultivation, using four commercial hybrids of melon (*Cucumis melo* L.), 2001 and 2003 (Italian netted, short shelf-life—SSL type), 2005 (Amarillo type), and 2008 (Galia, long shelf-life—LSL type).

In the greenhouse, the experiment was conducted in a 36.48 m^2^ area (19.2 m × 1.9 m). Plants were arranged in 3 rows, each containing 1 replicate of the 4 hybrids (for a total of 20 plants per hybrid). In the open field, the trial covered 192 m^2^ (38.4 m × 5.0 m), using 3 central rows (96 plants for area), each also hosting 1 replicate per treatment in a similar layout (24 plants per hybrid).

Seeds were sown on 9 February 2024 in 84-cell trays and pre-germinated for 72 h at 22.5 °C and 78% relative humidity; then, seedlings were transferred to a heated nursery. On 13 March, plants with 2 true leaves were manually transplanted into the greenhouse in twin rows with a resulting density of 2.2 plants m^−2^ (1.2 m inter-row, 0.7 m inter-twin, and 0.4 m intra-row spacing). In the open field, sowing and transplanting were performed on 26 April and 30 May 2024, respectively. The plants were arranged with a row spacing of 2.5 m and 0.8 m within the row, for a density of 0.5 plants m^−2^, according to the usual practice of the farm. Transplants were initially protected using mini-tunnels (0.80 m height, 0.60–0.70 m width), consisting of iron semicircles and covered with nonwoven geotextile, increasing the air temperature by more than 10 °C and soil temperature by 2–3 °C compared to outside. Tunnels were removed between 23 and 25 June to allow for plant acclimatation. Honeybee (*Apis mellifera*) hives were used to ensure adequate pollination.

In both environments, the soil was classified as sandy loam, with a pH of 6.8, organic matter content of 1.14%, total nitrogen (N) of 0.075%, phosphorus of 30 ppm, potassium of 314 ppm, and a C:N ratio of 8.8. Irrigation was managed according to the crop requirement in each environment, and fertigation was applied during the vegetative phase using a balanced N–P–K fertilizer enriched with micronutrients (involving 100 kg ha^−1^ of YaraTera Kristalon; Yara International ASA, Oslo, Norway). Foliar applications of 4 L per hectare of micronutrient-based fertilizers containing B and Mg were also carried out with Smart Quatro (Brandt Inc., Tampa, FL, USA). Once fruits reached approximately 8 cm in diameter, 3 additional fertigation events were performed with potassium sulphate to support fruit development, using Haifa MKP (Haifa Group, Haifa, Israel) at a dose of 35 kg ha^−1^. In the open field, post-transplant fertigation was supplemented with 150 kg ha^−1^ of calcium, magnesium, and ammonium nitrate (Ferti Ca/Mg; Fertenia s.r.l., Bellizzi, Italy) during vegetative growth. Following the removal of protective tunnels, 5 additional fertigation treatments (1 week apart) were applied to promote fruit growth and ripening (using the same fertilizer based on potassium sulphate above mentioned, at the same doses). In the greenhouse, the harvest started on 16 June and ended on 24 June, based on physiological maturity, assessed by peduncle detachment and days from fruit set. In the open field, the harvest was conducted from 28 July to 16 August, following the same criteria. In both cases, harvest was done progressively to match the fruit ripening rhythm. In both cultivation systems, the temperature and relative humidity (RH) were continuously monitored throughout the entire crop cycle using the automated PRIVA system (De Lier, The Netherlands). Daily mean values were calculated from hourly data (Figure 1). Greenhouse cultivation was conducted under natural solar radiation without artificial light supplementation, and crop development relied solely on ambient seasonal photosynthetically active radiation (PAR) typical of spring–early summer conditions.

### 4.2. Fruit Production and Quality Traits

At each harvest, plant productivity was assessed by recording the number of fruits per plant and the total yield, expressed in kg m^−2^. Fruits at physiological maturity were harvested daily, individually counted, cut, and weighed.

Total soluble solids (TSS) were measured in the juice of each fruit using a portable optical refractometer with automatic temperature compensation (ATC Refractometer, Brix range 0–32%, Giorgio Bormac s.r.l., Carpi, Italy), and values were expressed as °Brix. For each treatment (hybrid per cultivation environment), fresh biomass was determined on one representative fruit per replicate (3 fruits in total per treatment). Each fruit was divided into two sub-samples: one was immediately frozen in liquid nitrogen, lyophilized, and stored at –80 °C for future phytochemical analyses, and the other was used to determine the water content by oven drying at 70 °C until a constant weight (~72 h). Dried tissues were ground to a particle size of 0.5 mm using a cutting-head mill (IKA MF 10.1, Staufen, Germany) for subsequent mineral content analysis.

At the end of the cultivation cycle, the aboveground plant biomass, including stems and leaves, was harvested and weighed to determine the fresh weight. Plant samples were then dried in craft paper bags (40 × 60 cm) at 65 °C for 48 h to determine the dry matter content.

### 4.3. SPAD Index Determination

SPAD index measurement provides an indirect estimate of the leaf chlorophyll content, which is closely associated with the photosynthetic activity and tissue nitrogen concentration [28]. SPAD values are commonly used to assess the crop nutritional status under field conditions [3]. Measurements were taken using a SPAD-502 Plus chlorophyll meter (Minolta Corp. Ltd., Osaka, Japan). The instrument measures light transmittance through leaves at two wavelengths: ~650 nm (red, high chlorophyll absorbance) and ~930 nm (infrared, low absorbance) [43]. Light passing through the leaf is captured by a photodiode and converted into an electric signal, which is processed into a SPAD unit. SPAD readings were conducted during fruit set on well-developed leaves [44]. Measurements were taken from three different points on the fourth leaf from the apical meristem: the base, middle, and tip of the leaf. These points were chosen to capture the variability in the chlorophyll content along the leaf blade, as the chlorophyll distribution can differ between the proximal and distal regions of the leaf. Data were recorded in Microsoft Excel for subsequent statistical analysis.

### 4.4. Multi Elemental Profile Evaluation

The total carbon (C), nitrogen (N), and sulfur (S) concentrations were measured in freeze-dried, powdered samples (2 mg) using a Micro Elemental Analyzer (UNICUBE^®^, Elementar, Germany), calibrated with sulphanilamide (99.5%) for quality control. The total concentrations of Ca, P, K, Mg, Na, Fe, Mn, Zn, Cu, and B were determined by ICP-MS (Thermo Scientific iCAP Q, USA) following microwave-assisted acid digestion of 100 mg freeze-dried samples. The digestion employed a mixture of 65% HNO_3_ (2.5 mL), 3 M HCl (0.5 mL), and 50% HF (0.5 mL). Analytical accuracy was verified using certified reference material NCS ZC85006, with element recoveries within ±10% of certified values.

### 4.5. Polyphenolic Profile Assessment

Extraction of phenolic compounds from melon samples followed the method of Rodríguez-Pérez et al. [41] with slight modifications. Briefly, 0.5 g of freeze-dried melon was extracted with 5 mL of methanol: water (80:20, v:v). The mixture was vortexed for 3 min, sonicated for 15 min, agitated on a rotary shaker for 10 min, and centrifuged at 5000× *g* for 5 min at 4 °C. The supernatant was collected, and the residue was re-extracted using the same procedure. Combined supernatants were filtered through a 0.2 μm nylon membrane and diluted with methanol containing 0.1% formic acid.

Chromatographic separation was performed on a UHPLC system (Thermo Fisher Scientific, Waltham, MA, USA) using a Dionex Ultimate 3000 pump and a Kinetex 1.7 μm biphenyl column (100 × 2.1 mm, Phenomenex) at 25 °C. A 2 μL injection was run at 0.2 mL/min with a gradient of 0.1% formic acid in water (A) and methanol (B): 0 min, 5% B; 1.3 min, 30% B; 9.3 min, 100% B; 11.3 min, 100% B; 13.3 min, 5% B; 20 min, 5% B [45].

Mass spectrometry was conducted using a Q Exactive Orbitrap LC-MS/MS (Thermo Fisher Scientific, Waltham, MA, USA) in Full MS mode with a resolution of 70,000 FWHM and mass tolerance of 5 ppm. Targeted analysis employed Parallel Reaction Monitoring (PRM) with optimized retention times and collision energies for each polyphenol. Method linearity was assessed between 5 and 120 mg/kg using 6 calibration points. Limits of detection (LOD) and quantification (LOQ) were established based on signal-to-noise ratios of chlorogenic acid and rutin standards via serial dilutions. Data analysis was performed using Xcalibur software (v. 3.1.66.19).

### 4.6. Experimental Design and Statistical Analysis

Data were subjected to two-way analysis of variance (ANOVA) using IBM SPSS Statistics, version 2022 (IBM Corp., Armonk, NY, USA). The experimental design included two factors: cultivation system (factor S), comparing open-field horizontal training versus greenhouse vertical training, and hybrid (factor H), consisting of four commercial melon genotypes. Main effects were assessed using Student’s t-test for factor A and one way ANOVA for factor C. When significant differences were detected (*p* < 0.05), means were compared using the Tukey–Kramer HSD post hoc test for factor C and the A × C interaction. A significance level of *p* < 0.05 was used throughout the analysis.

## 5. Conclusions

Our study demonstrated that the cultivation system and plant genotype significantly affect both the fruit production and the nutritional–nutraceutical quality of fruits in melon (*Cucumis melo* L.). Vertical cultivation under greenhouse conditions was effective in optimizing the space use efficiency and increasing the yield per unit area, while enabling earlier production and offering the potential for more intensive cropping schedules compared to open-field cultivation. This approach is especially advantageous for intensive or small-scale farming operations. Notably, fruits produced in the greenhouse exhibited high concentrations of flavonoids and vanillic acid, thereby enhancing their nutraceutical profile and potential health-promoting properties.

Among the evaluated hybrids, genotypes 2005 and 2008 demonstrated higher yield and soluble solids contents across both cultivation systems, underscoring their adaptability and agronomic potential. The observed genetic variability among the hybrids further highlights the importance of targeted genotype selection in relation to the production system. Such strategic matching between the genotype and cultivation method can contribute to achieving desired agronomic performance, fruit quality, and market alignment.

Overall, these findings provide a valuable basis for breeding programs and grower decision-making, supporting the selection of melon hybrids best suited to specific environmental conditions and production systems. The integration of genotype-specific responses with optimized cultivation strategies can contribute to the sustainable production of melon, ensuring both high yield and improved fruit quality.

In conclusion, the individual hybrid results reveal a noteworthy degree of genetic variability, underscoring the critical importance of matching varietal selection to the cultivation environment. Such alignment can enhance the plant’s adaptability to the cropping system and ultimately help producers achieve improved yield and fruit quality. Future research could focus on investigating additional hybrid combinations under different vertical farming conditions in order to enhance both plant performance and crop management strategies for multiple harvest cycles and to explore the physiological mechanisms underlying the adaptability to high-density cropping systems. Such studies will help further improve efficiency, productivity, and sustainability in controlled-environment agriculture.

## Figures and Tables

**Table 1:** Growth parameters in the four tested hybrids, 2001, 2003, 2005, and 2008, grown in the two cultivation systems, open field with trailing growth (FT) and greenhouse with vertical training (GV).

Treatment	Leaf Greenness ^1^ SPAD Units	Fresh Weight ^2^ kg m^−2^	Dry Weight ^2^ kg m^−2^	Dry Matter Content ^2^ %
Cultivation system (S)				
FT	97.5 ± 2.6	3.62 ± 0.07	0.80 ± 0.02	21.3 ± 0.70
GV	89.4 ± 4.2	0.81 ± 0.03	0.08 ± 0.00	10.4 ± 0.31
Hybrid (H)				
2001	81.4 ± 6.9 b	2.44 ± 0.58 a	0.40 ± 0.12 c	13.9 ± 1.49 c
2003	87.7 ± 2.0 b	2.18 ± 0.53 c	0.43 ± 0.13 b	15.3 ± 1.86 bc
2005	103 ± 2.2 a	1.95 ± 0.48 d	0.43 ± 0.13 b	16.8 ± 2.49 ab
2008	102 ± 3.1 a	2.30 ± 0.54 b	0.52 ± 0.16 a	17.3 ± 2.60 a
S × H				
FT × 2001	98.7 ± 3.6 abc	3.97 ± 0.01 a	0.70 ± 0.00 c	17.7 ± 0.12 b
FT × 2003	83.1 ± 1.3 d	3.57 ± 0.02 b	0.78 ± 0.01 b	20.1 ± 0.57 b
FT × 2005	99.7 ± 3.2 abc	3.22 ± 0.02 c	0.78 ± 0.00 b	23.4 ± 0.50 a
FT × 2008	108 ± 1.5 a	3.74 ± 0.02 b	0.96 ± 0.01 a	24.2 ± 0.34 a
GV × 2001	64.1 ± 3.0 e	0.91 ± 0.03 d	0.09 ± 0.01 d	10.2 ± 1.06 c
GV × 2003	92.4 ± 1.6 cd	0.79 ± 0.09 de	0.08 ± 0.01 d	10.5 ± 0.69 c
GV × 2005	106 ± 2.5 ab	0.69 ± 0.04 e	0.07 ± 0.00 d	10.3 ± 0.39 c
GV × 2008	95.0 ± 3.4 bcd	0.86 ± 0.04 de	0.09 ± 0.00 d	10.5 ± 0.41 c
Cultivation system (S)	ns	***	***	***
Hybrid (H)	***	***	***	***
S × H	***	***	***	***

^1^ SPAD measurements were taken at the fruit set stage on fully expanded leaves (three positions on the fourth leaf from the vegetative apex). ^2^ All other measurements were performed at the end of the crop cycle. ns, ***: Not significant or significant difference at *p* ≤ 0.01 and 0.001, respectively. Different letters indicate values significantly separated according to Tukey’s multiple comparison test (*p* = 0.05).

**Table 2:** Yield components in the four tested hybrids, 2001, 2003, 2005, and 2008, grown in the two cultivation systems, open field with trailing growth (FT) and greenhouse with vertical training (GV).

Treatment	Number of Fruits (n plant^−1^)	Fruit Weight (kg fruit^−1^)	Fruit Yield (kg m^−2^)	Pulp Dry Matter (%)	Refractometric Index (° Brix)
Cultivation system (S)					
FT	7.00 ± 0.21 a	1.81 ± 0.05 a	6.58 ± 0.36 b	11.27 ± 0.27	12.73 ± 0.34
GV	2.81 ± 0.14 b	1.18 ± 0.03 b	7.77 ± 0.41 a	11.09 ± 0.58	12.88 ± 0.59
Hybrid (H)					
2001	5.13 ± 0.77 ab	1.30 ± 0.10 b	6.30 ± 0.44 b	10.75 ± 0.36 b	10.93 ± 0.15 c
2003	4.63 ± 0.80 bc	1.52 ± 0.16 a	6.71 ± 0.51 b	9.44 ± 0.54 c	11.54 ± 0.30 c
2005	4.31 ± 0.74 c	1.62 ± 0.12 a	7.02 ± 0.36 ab	13.33 ± 0.37 a	15.31 ± 0.39 a
2008	5.56 ± 0.94 a	1.53 ± 0.11 a	8.67 ± 0.66 a	11.18 ± 0.22 b	13.44 ± 0.21 b
S × H					
FT × 2001	7.13 ± 0.24	1.53 ± 0.05 b	5.69 ± 0.70	11.24 ± 0.52 bc	11.02 ± 0.26 c
FT × 2003	6.63 ± 0.47	1.94 ± 0.04 a	6.57 ± 0.72	10.54 ± 0.49 c	12.06 ± 0.19 c
FT × 2005	6.25 ± 0.14	1.93 ± 0.06 a	6.67 ± 0.56	12.46 ± 0.35 ab	14.38 ± 0.18 b
FT × 2008	8.00 ± 0.20	1.82 ± 0.02 a	7.40 ± 0.90	10.83 ± 0.32 bc	13.44 ± 0.20 b
GV × 2001	3.13 ± 0.13	1.06 ± 0.05 d	6.91 ± 0.41	10.26 ± 0.42 c	10.84 ± 0.18 c
GV × 2003	2.63 ± 0.31	1.10 ± 0.02 d	6.85 ± 0.81	8.35 ± 0.58 d	11.01 ± 0.45 c
GV × 2005	2.38 ± 0.24	1.31 ± 0.05 c	7.37 ± 0.45	14.20 ± 0.16 a	16.24 ± 0.31 a
GV × 2008	3.13 ± 0.31	1.24 ± 0.04 cd	9.94 ± 0.39	11.54 ± 0.19 bc	13.45 ± 0.41 b
Cultivation system (S)	***	***	*	ns	ns
Hybrid (H)	***	***	**	***	***
S × H	ns	**	ns	***	***

ns, *, **, ***: Not significant or significant differences at *p* ≤ 0.05, 0.01, and 0.001, respectively. Different letters indicate significantly different values according to Tukey’s multiple comparison test (*p* ≤ 0.05).

**Table 3:** Contents of macroelements, carbon, and sodium (g kg^−1^) in melon fruit pulp of the four tested hybrids, 2001, 2003, 2005, and 2008, grown in the two cultivation systems, open field with trailing growth (FT) and greenhouse with vertical training (GV).

Treatment	C	K	N	S	P	Ca	Mg	Na
Cultivation system (S)								
FT	419.5 ± 1	32.8 ± 1.3	14.6 ± 0.5	3.75 ± 0.56	1.51 ± 0.09	1.02 ± 0.1	1.14 ± 0.05	0.56 ± 0.04
GV	422.4 ± 1.6	35.6 ± 2	18.3 ± 1.0	3.88 ± 0.56	3.29 ± 0.36	0.9 ± 0.14	1.52 ± 0.17	1.33 ± 0.11
Hybrid (H)								
2001	419.5 ± 1.3 b	38 ± 1.8 a	17.8 ± 1.2 a	5.19 ± 0.92	2.34 ± 0.31 b	1.29 ± 0.13 a	1.65 ± 0.21 a	0.81 ± 0.14 ab
2003	418.2 ± 2.1 b	40.7 ± 1.6 a	19.9 ± 1.3 a	4.12 ± 0.86	3.76 ± 0.68 a	1.38 ± 0.09 a	1.72 ± 0.18 a	0.79 ± 0.11 b
2005	427 ± 1.5 a	26.5 ± 1 c	14.4 ± 0.8 b	2.45 ± 0.2	1.84 ± 0.22 c	0.47 ± 0.07 b	1.01 ± 0.06 b	1.21 ± 0.2 a
2008	419.2 ± 1.1 b	31.6 ± 1 b	13.6 ± 0.4 b	3.5 ± 0.69	1.65 ± 0.19 c	0.69 ± 0.1 b	0.92 ± 0.07 b	0.96 ± 0.23 ab
S × H								
FT × 2001	417.9 ± 2.1	35.5 ± 1.9	15 ± 1.0 b	4.72 ± 1.19	1.55 ± 0.14 de	1.16 ± 0.14	1.21 ± 0.11 b	0.47 ± 0.04
FT × 2003	418.2 ± 2.3	37.5 ± 2.1	16.8 ± 0.9 b	5.27 ± 1.6	1.95 ± 0.06 cd	1.42 ± 0.15	1.26 ± 0.08 b	0.55 ± 0.05
FT × 2005	423.8 ± 1.4	27.5 ± 1.9	13.2 ± 0.8 b	2.79 ± 0.31	1.33 ± 0.08 e	0.61 ± 0.1	1.11 ± 0.11 b	0.78 ± 0.03
FT × 2008	418.1 ± 1.3	30.8 ± 1.6	13.2 ± 0.6 b	2.22 ± 0.03	1.2 ± 0.07 e	0.87 ± 0.06	0.96 ± 0.08 b	0.44 ± 0.07
GV × 2001	421.1 ± 1.3	40.5 ± 2.7	20.7 ± 0.7 a	5.66 ± 1.55	3.13 ± 0.09 b	1.42 ± 0.21	2.1 ± 0.26 a	1.16 ± 0.1
GV × 2003	418.2 ± 3.9	43.9 ± 1.1	23 ± 0.7 a	2.98 ± 0.08	5.57 ± 0.03 a	1.34 ± 0.13	2.19 ± 0.08 a	1.03 ± 0.14
GV × 2005	430.1 ± 1.2	25.4 ± 0.7	15.5 ± 1.1 b	2.11 ± 0.08	2.36 ± 0.2 c	0.33 ± 0.01	0.91 ± 0.01 b	1.64 ± 0.25
GV × 2008	420.4 ± 1.9	32.4 ± 1.3	14 ± 0.4 b	4.78 ± 1.07	2.09 ± 0.15 c	0.51 ± 0.13	0.88 ± 0.13 b	1.48 ± 0.27
Cultivation system (S)	ns	ns	**	ns	***	ns	*	***
Hybrid (H)	***	***	***	ns	***	***	***	*
S × H	ns	ns	**	ns	***	ns	***	ns

ns, *, **, ***: Not significant or significant at *p* ≤ 0.05, 0.01, and 0.001, respectively. Different letters indicate values that are significantly different according to Tukey’s multiple comparison test (*p* = 0.05).

**Table 4:** Contents of microelements (ppm) in the pulp of melon fruits of the four tested hybrids, 2001, 2003, 2005, and 2008, grown in the two cultivation systems, open field with trailing growth (FT) and greenhouse with vertical training (GV).

Treatment	Fe	Mn	B	Zn	Cu	Mo	Se
Cultivation system (S)							
FT	25.5 ± 0.94	6.36 ± 0.52	12.1 ± 0.49	12.07 ± 0.88	4.8 ± 0.2	0.069 ± 0.024	0.020 ± 0.002
VT	30.4 ± 1.89	6.98 ± 0.82	17.7 ± 1.12	15.47 ± 1.99	3.92 ± 0.3	0.183 ± 0.021	0.025 ± 0.003
Hybrid (H)							
2001	32 ± 2.72 a	7.69 ± 1.05 ab	15.7 ± 1.2 b	19.17 ± 1.8 a	5.47 ± 0.21 a	0.139 ± 0.036 b	0.029 ± 0.003 ab
2003	31.5 ± 1.67 a	9.38 ± 0.67 a	18.5 ± 2.03 a	17.59 ± 2.47 a	4.55 ± 0.39 b	0.098 ± 0.030 bc	0.030 ± 0.004 a
2005	21.6 ± 0.74 b	4.09 ± 0.3 c	11.2 ± 0.55 c	9.38 ± 0.39 b	3.83 ± 0.26 bc	0.044 ± 0.016 c	0.018 ± 0.002 bc
2008	26.6 ± 1.31 ab	5.51 ± 0.38 bc	14.1 ± 1.06 b	8.96 ± 0.27 b	3.6 ± 0.32 c	0.225 ± 0.036 a	0.016 ± 0.002 c
S × H							
FT × 2001	28 ± 2.01	5.8 ± 0.61 bc	13.5 ± 1.19 cd	15.12 ± 0.53	5.27 ± 0.28 a	0.057 ± 0.027	0.024 ± 0.005
FT × 2003	27.8 ± 1.33	8.9 ± 1.01 ab	13.4 ± 0.56 cd	15.5 ± 1.02	5.37 ± 0.45 a	0.024 ± 0.005	0.024 ± 0.003
FT × 2005	21.3 ± 0.93	4.42 ± 0.57 c	9.8 ± 0.33 d	8.51 ± 0.31	4.27 ± 0.2 ab	0.002 ± 0.003	0.016 ± 0.002
FT × 2008	24.6 ± 1.06	6.31 ± 0.41 abc	11.6 ± 0.16 d	9.16 ± 0.28	4.3 ± 0.38 ab	0.194 ± 0.058	0.018 ± 0.002
GV × 2001	36 ± 4.48	9.59 ± 1.53 ab	17.9 ± 1.42 b	23.22 ± 1.98	5.68 ± 0.3 a	0.221 ± 0.029	0.033 ± 0.003
GV × 2003	35.2 ± 1.48	9.85 ± 0.98 a	23.5 ± 1.3 a	19.67 ± 4.96	3.74 ± 0.26 b	0.172 ± 0.024	0.034 ± 0.008
GV × 2005	21.9 ± 1.27	3.75 ± 0.12 c	12.5 ± 0.34 d	10.24 ± 0.32	3.38 ± 0.36 b	0.085 ± 0.006	0.020 ± 0.002
GV × 2008	28.5 ± 2.1	4.72 ± 0.29 c	16.7 ± 0.87 bc	8.76 ± 0.5	2.91 ± 0.13 b	0.255 ± 0.044	0.014 ± 0.003
Cultivation system (S)	*	ns	***	ns	*	**	ns
Hybrid (H)	***	***	***	***	***	***	**
S × H	ns	*	**	ns	*	ns	ns

ns, *, **, ***: Not significant or significant at *p* ≤ 0.05, 0.01, and 0.001, respectively. Different letters indicate values that are significantly different according to Tukey’s multiple comparison test (*p* = 0.05).

**Table 5:** Contents of macroelements, carbon, and sodium (g kg^−1^) in the peel of melon fruits in the four tested hybrids, 2001, 2003, 2005, and 2008, grown in the two cultivation systems, open field with trailing growth (FT) and greenhouse with vertical training (GV).

Treatment	C	K	N	S	P	Ca	Mg	Na
Cultivation system (S)								
FT	411 ± 2.07	47.7 ± 1.06	21.76 ± 1.04	4.59 ± 0.5	3.74 ± 0.16	6.56 ± 0.31	2.78 ± 0.08	1.36 ± 0.08
GV	413 ± 4.02	41 ± 1.33	22.79 ± 0.8	4.23 ± 0.39	6.98 ± 0.29	5.44 ± 0.39	3.92 ± 0.17	2.71 ± 0.2
Hybrid (H)								
2001	418 ± 6.27	45.2 ± 1.29 a	24.15 ± 1.04 a	4.55 ± 0.64	5.08 ± 0.62 b	6.1 ± 0.3	3.26 ± 0.24 ab	1.62 ± 0.23 b
2003	408 ± 3.95	45.7 ± 1.25 a	22.04 ± 0.9 ab	3.39 ± 0.13	5.26 ± 0.67 b	6.59 ± 0.52	3.79 ± 0.38 a	1.83 ± 0.24 ab
2005	417 ± 3.36	39.8 ± 2.85 b	18.53 ± 1.26 b	4.3 ± 0.75	4.82 ± 0.48 b	4.96 ± 0.67	3.18 ± 0.23 b	2.53 ± 0.37 a
2008	405 ± 2.16	46.8 ± 1.74 a	24.4 ± 0.92 a	5.39 ± 0.67	6.3 ± 0.84 a	6.33 ± 0.46	3.16 ± 0.21 b	2.15 ± 0.38 ab
S × H								
FT × 2001	410 ± 4.64	47.2 ± 1.8	22.8 ± 1.87	5.36 ± 1.18	3.53 ± 0.24 c	6.33 ± 0.35	2.64 ± 0.11 c	1.02 ± 0.08
FT × 2003	412 ± 5.73	47.9 ± 1.68	20 ± 0.92	3.66 ± 0.11	3.6 ± 0.35 c	6.38 ± 0.98	2.82 ± 0.16 c	1.38 ± 0.13
FT × 2005	415 ± 4.51	45.8 ± 2.65	18.9 ± 2.66	3.37 ± 0.2	3.73 ± 0.46 c	6.41 ± 0.73	2.87 ± 0.21 bc	1.7 ± 0.15
FT × 2008	409 ± 1.71	49.9 ± 2.52	25.35 ± 1.27	5.96 ± 1.37	4.12 ± 0.2 c	7.09 ± 0.47	2.77 ± 0.15 c	1.35 ± 0.11
GV × 2001	426 ± 10.8	43.3 ± 1.39	25.5 ± 0.6	3.75 ± 0.22	6.63 ± 0.35 b	5.88 ± 0.5	3.87 ± 0.08 ab	2.23 ± 0.05
GV × 2003	404 ± 5.34	43.4 ± 1.06	24.08 ± 0.38	3.12 ± 0.12	6.91 ± 0.4 ab	6.8 ± 0.52	4.77 ± 0.18 a	2.29 ± 0.35
GV × 2005	418 ± 5.54	33.8 ± 2.6	18.15 ± 0.5	5.23 ± 1.42	5.91 ± 0.28 b	3.52 ± 0.42	3.48 ± 0.37 bc	3.35 ± 0.38
GV × 2008	402 ± 3.36	43.8 ± 1.3	23.45 ± 1.33	4.81 ± 0.13	8.48 ± 0.34 a	5.56 ± 0.61	3.56 ± 0.28 bc	2.95 ± 0.47
Cultivation system (S)	ns	***	ns	ns	***	*	***	***
Hybrid (H)	ns	**	***	ns	***	ns	*	*
S × H	ns	ns	ns	ns	*	ns	*	ns

ns, *, **, ***: Not significant or significant at *p* ≤ 0.05, 0.01, and 0.001, respectively. Different letters indicate values that are significantly different according to Tukey’s multiple comparison test (*p* = 0.05).

**Table 6:** Contents of microelements (ppm) in the peel of melon fruits in the four tested hybrids, 2001, 2003, 2005, and 2008, grown in the two cultivation systems, open field with trailing growth (FT) and greenhouse with vertical training (GV).

Treatment	Fe	Mn	B	Zn	Cu	Mo	Se
Cultivation system (S)							
FT	62.1 ± 2.61	16.6 ± 0.88	25.8 ± 0.6	17.3 ± 1.13	7.36 ± 0.71	0.070 ± 0.020	0.024 ± 0.002
GV	66 ± 3.79	79.7 ± 7.81	30.2 ± 1.26	27.3 ± 2.5	4.83 ± 0.37	0.695 ± 0.128	0.033 ± 0.002
Hybrid (H)							
2001	79.1 ± 4.27 a	47.5 ± 13.8 ab	26.8 ± 0.81 b	29.4 ± 2.54 a	8.39 ± 0.9 a	0.355 ± 0.098 b	0.030 ± 0.003 a
2003	61.1 ± 3.1 b	61.6 ± 18 a	32.1 ± 2.06 a	22.1 ± 4.58 ab	5.84 ± 0.51 b	0.236 ± 0.091 b	0.026 ± 0.002 a
2005	54.1 ± 3.11 b	33 ± 5.74 b	24.9 ± 0.84 b	19.7 ± 2.72 b	4.47 ± 0.58 b	0.173 ± 0.062 b	0.024 ± 0.002 b
2008	61.9 ± 2.61 b	50.6 ± 15.1 ab	28.2 ± 1.19 b	18.1 ± 1.05 b	5.67 ± 1.05 b	0.766 ± 0.273 a	0.034 ± 0.004 a
S × H							
FT × 2001	71.3 ± 4.15	16.2 ± 0.84 c	25.7 ± 1.03 b	23.3 ± 1.35	9.96 ± 1.39	0.117 ± 0.063 bc	0.026 ± 0.005
FT × 2003	62.4 ± 3.82	14.6 ± 2.73 c	26.9 ± 1.08 b	15.9 ± 1.36	6.74 ± 0.79	0.030 ± 0.005 c	0.021 ± 0.002
FT × 2005	50.6 ± 4.26	18.3 ± 2.14 c	24.5 ± 1.24 b	13.1 ± 1.68	4.95 ± 1.04	0.016 ± 0.006 c	0.023 ± 0.004
FT × 2008	64.2 ± 3.56	17.3 ± 0.5 c	26.3 ± 1.56 b	16.9 ± 0.97	7.77 ± 1.48	0.118 ± 0.027 bc	0.028 ± 0.004
GV × 2001	87 ± 5.17	78.7 ± 15.5 ab	27.9 ± 1.06 b	35.6 ± 1.69	6.83 ± 0.5	0.592 ± 0.060 b	0.035 ± 0.004
GV × 2003	59.7 ± 5.38	108.6 ± 5.52 a	37.3 ± 0.85 a	28.3 ± 8.39	4.94 ± 0.23	0.441 ± 0.103 bc	0.031 ± 0.002
GV × 2005	57.6 ± 4.36	47.7 ± 2.29 bc	25.3 ± 1.28 b	26.2 ± 1.8	3.99 ± 0.58	0.331 ± 0.040 bc	0.026 ± 0.002
GV × 2008	59.6 ± 3.94	84 ± 17.8 ab	30.1 ± 1.34 b	19.3 ± 1.79	3.58 ± 0.23	1.414 ± 0.261 a	0.040 ± 0.004
Cultivation system (S)	ns	***	**	***	**	***	**
Hybrid (H)	***	*	***	*	**	***	*
S × H	ns	*	**	ns	ns	***	ns

ns, *, **, ***: Not significant or significant at *p* ≤ 0.05, 0.01, and 0.001, respectively. Different letters indicate values that are significantly different according to Tukey’s multiple comparison test (*p* = 0.05).

**Table 7:** Polyphenol content (µg g^−1^) in the pulp of four melon hybrids (2001, 2003, 2005, and 2008), grown under two cultivation systems: open field with trailing growth (FT) and greenhouse with vertical training (GV).

Treatment	Phenolic Acid	Flavonoids	Vanillic Acid	Total Phenols
Cultivation system (S)				
FT	22.75 ± 2.74	0.37 ± 0.11	55.83 ± 6.28	68.48 ± 6.87
GV	19.3 ± 2.24	11.37 ± 3.94	53.12 ± 7.2	83.79 ± 11.5
Hybrid (H)				
2001	30.9 ± 3.19 a	11.64 ± 7.44	73.78 ± 5.64 a	107.1 ± 13.34 a
2003	24.97 ± 2.9 ab	9.1 ± 3.37	63.84 ± 7.75 a	89.94 ± 11.72 a
2005	14.79 ± 1.17 b	0.15 ± 0.03	26.13 ± 7.83 b	37.79 ± 7.41 c
2008	13.44 ± 2.34 b	2.59 ± 2.21	53.69 ± 7.73 a	69.72 ± 7.26 b
S × H				
FT × 2001	32.77 ± 5.39	0.42 ± 0.17	63.81 ± 2.98 a	81.05 ± 14.01 bc
FT × 2003	25.19 ± 6.1	0.31 ± 0.2	51.91 ± 12.04 ab	64.43 ± 11.05 cd
FT × 2005	16.81 ± 1.51	0.08 ± 0.03	38.2 ± 17.16 ab	45.54 ± 14.63 cd
FT × 2008	16.22 ± 4.43	0.66 ± 0.37	66.02 ± 12.51 a	82.9 ± 10.38 abc
GV × 2001	29.04 ± 3.99	22.86 ± 13.21	81.26 ± 7.96 a	133.15 ± 13.46 a
GV × 2003	24.76 ± 1.35	17.89 ± 1.13	72.79 ± 8.69 a	115.44 ± 9.24 ab
GV × 2005	12.76 ± 1.16	0.21 ± 0.04	17.08 ± 1.93 b	30.05 ± 1.53 d
GV × 2008	10.65 ± 0.82	4.53 ± 4.48	41.36 ± 4.56 ab	56.54 ± 4.7 cd
Cultivation system (S)	ns	**	ns	ns
Hybrid (H)	***	ns	***	***
S × H	ns	ns	*	***

ns, *, **, ***: Not significant or significant at *p* ≤ 0.05, 0.01, and 0.001, respectively. Different letters indicate values that are significantly different according to Tukey’s multiple comparison test (*p* = 0.05).

## Data Availability

The raw data supporting the conclusions of this article will be made available by the authors, without undue reservation.

## Abbreviations

TSS, total soluble solids; RDA, recommended daily allowance; LSL, long shelf life; SSL, short shelf life; MS, mass spectrometry.

## References

[1] Zhang G., Li Z., Liu L., Xiang Q. (2024). Road to valorisation of melon seeds (*Cucumis melo* L.): A comprehensive review of nutritional profiles, biological activities, and food applications. Sustain. Food Technol..

[2] Manchali S., Chidambara Murthy K.N., Vishnuvardana, Patil B.S. (2021). Nutritional composition and health benefits of various botanical types of melon (*Cucumis melo* L.). Plants.

[3] Shah I.H., Jinhui W., Ding X., Li X., Rehman A., Azam M., Chang L. (2025). A non-destructive approach: Estimation of melon fruit quality attributes and nutrients using hyperspectral imaging coupled with machine learning. Smart Agric. Technol..

[4] Schemberger M.O., Stroka M.A., Reis L., de Souza Los K.K., de Araujo G.A.T., Sfeir M.Z.T., Ayub R.A. (2020). Transcriptome profiling of non-climacteric ‘yellow’ melon during ripening: Insights on sugar metabolism. BMC Genom..

[5] Stroka M.A., Reis L., de Souza Los K.K., Pinto C.A., Gustani F.M., Forney C.F., Ayub R.A. (2024). The maturation profile triggers differential expression of sugar metabolism genes in melon fruits. Plant Physiol. Biochem..

[6] Lestari N.D., Handayani N. (2025). The effect of variation of species and types of sugar on the quality of fruitghurt from the mesocarp layer of watermelon (*Citrullus lanatus*) based on organoleptic tests. BIO Web Conf..

[7] Seo M.H., Tilahun S., Melaku A., Jeong C.S. (2018). Effect of ripening conditions on the quality and storability of muskmelon (*Cucumis melo* L.) fruits. Hortic. Sci. Technol..

[8] Sultana H., Mallick S.R., Hassan J., Gomasta J., Kabir M.H., Sakib M.S.A., Kayesh E. (2023). Nutritional composition and bioactive compounds of mini watermelon genotypes in Bangladesh. arXiv.

[9] Ezzat S.M., Raslan M., Salama M.M., Menze E.T., El Hawary S.S. (2019). In vivo anti-inflammatory activity and UPLC-MS/MS profiling of the peels and pulps of *Cucumis melo* var. *cantalupensis* and *Cucumis melo* var. reticulatus. J. Ethnopharmacol..

[10] Di Sotto A., Di Giacomo S. (2023). Plant polyphenols and human health: Novel findings for future therapeutic developments. Nutrients.

[11] Evana E., Barek M.S. (2021). Determination of vitamin C (ascorbic acid) contents in two varieties of melon fruits (*Cucumis melo* L.) by iodometric titration. Fuller. J. Chem..

[12] Gómez-García R., Campos D.A., Oliveira A., Aguilar C.N., Madureira A.R., Pintado M. (2021). A chemical valorisation of melon peels towards functional food ingredients: Bioactives profile and antioxidant properties. Food Chem..

[13] Mallek-Ayadi S., Bahloul N., Baklouti S., Kechaou N. (2022). Bioactive compounds from *Cucumis melo* L. fruits as potential nutraceutical food ingredients and juice processing using membrane technology. Food Sci. Nutr..

[14] Farcuh M., Copes B., Le-Navenec G., Marroquin J., Cantu D., Bradford K.J., Van Deynze A. (2020). Sensory, physico-chemical and volatile compound analysis of short and long shelf-life melon (*Cucumis melo* L.) genotypes at harvest and after postharvest storage. Food Chem. X.

[15] FAOSTAT FAO Statistical Databases. Food and Agriculture Organization of the United Nations. https://www.fao.org/faostat/en/#home.

[16] ISTAT. Italian National Institute of Statistics. https://www.istat.it/en/.

[17] Pethybridge S., Damann K., Murphy S., Diggins K.R., Gleason M.L. (2024). Optimizing organic muskmelon production by integrating mesotunnel row covers, inter-bed weed management, and pollination strategies. Renew. Agric. Food Syst..

[18] Adamović B., Vojnović Đ., Ilin Ž. (2024). Distinctive aspects of early open-field watermelon production. AIDASCO Rev..

[19] Amaroek S., Jansaku W., Panton S., Rakyat M., Namwong P., Janjenjob S., Jinda B.N. (2025). Development of suitable greenhouse to increase melon production efficiency. Agric. Biol. Eng..

[20] Zhuang Y., Zhang C.Y., Lu N. (2025). Environmental performance of fruiting vegetable production in vertical farms. Sustain. Prod. Consum..

[21] Singh A.K., Singh J., Jat G. (2025). Vertical and terraced farming for higher vegetable production. Indian Farming.

[22] McCreight J.D., Salinas C.A., Davis A.A., Woodland C.A., Reitsma K. (2020). Melon Crop Vulnerability Statement 2020.

[23] Maniçoba F.E., Negreiros A.M.P., Cavalcante A.L.A., Santos Alves C.P.D.S., Nascimento M.T.D.A.E., Ambrósio M.M.D.Q., Sales Júnior R. (2023). Effect of environmental factors, fungicide sensitivity, and pathogenicity of *Fusarium* spp. associated with fruit rot of melon. J. Phytopathol..

[24] Jain S., Kore D.S., GK K., Mohapatra A., Baksh H., Kumar V., Haokip S.W. (2023). A comprehensive review on protected cultivation of horticultural crops: Present status and future prospects. Int. J. Environ. Clim. Change.

[25] Battistel P. (2014). Melone coltivato in verticale. Colture Protette.

[26] Gruda N.S., Gallegos-Cedillo V.M., Nájera C., Catalina E.G., Ochoa J., Fernández J.A. (2025). Advancing protected cultivation: A pathway for nutrient-rich vegetables. Crit. Rev. Plant Sci..

[27] Weng J., Rehman A.U., Li P., Chang L., Zhang Y., Niu Q. (2022). Physiological and transcriptomic analysis reveals the responses and difference to high temperature and humidity stress in two melon genotypes. Int. J. Mol. Sci..

[28] Peterson T., Blackmer T., Francis D., Scheppers J. (1993). Using a chlorophyll meter to improve N management. Soil Resource Management Web Guide D-13 Fertility.

[29] Vescera M., Brown R. (2016). Effects of Three Production Systems on Muskmelon Yield and Quality in New England. Hort. Sci..

[30] Vatistas C., Avgoustaki D.D., Bartzanas T. (2022). A systematic literature review on controlled-environment agriculture: How vertical farms and greenhouses can influence the sustainability and footprint of urban microclimate with local food production. Atmosphere.

[31] Dong T., Shang J., Chen J.M., Liu J., Qian B., Ma B., Zhou G. (2019). Assessment of portable chlorophyll meters for measuring crop leaf chlorophyll concentration. Remote Sens..

[32] Amarasinghe R.M., Sakimin S.Z., Wahab P.E., Ramlee S., Nakasha J.J. (2021). Growth, physiology and yield responses of four rock melon (*Cucumis melo* var. *cantaloupensis*) cultivars in elevated temperature. Plant Arch..

[33] Ríos P., Argyris J., Vegas J., Leida C., Kenigswald M., Tzuri G., García-Más J. (2017). *ethqv6.3* is involved in melon climacteric fruit ripening and is encoded by a NAC domain transcription factor. Plant J..

[34] Pscheidt J.W., Ocamb C.M. (2022). Copper-based bactericides and fungicides. Pacific Northwest Pest Management Handbooks.

[35] Hsieh E., Waters B.M. (2016). Alkaline stress and iron deficiency regulate iron uptake and riboflavin synthesis gene expression differently in root and leaf tissue: Implications for iron deficiency chlorosis. J. Exp. Bot..

[36] Durazzo A., Lucarini M., Souto E.B., Cicala C., Caiazzo E., Izzo A.A., Novellino E., Santini A. (2019). Polyphenols: A concise overview on the chemistry, occurrence, and human health. Phytother. Res..

[37] Wang Y., Gao H., Guo Z., Peng Z., Li S., Zhu Z., Grimi N., Xiao J. (2023). Free and bound phenolic profiles and antioxidant activities in melon (*Cucumis melo* L.) pulp: Comparative study on six widely consumed varieties planted in Hainan Province. Foods.

[38] Kolaylı S., Kara M., Tezcan F., Erim F.B., Şahin H., Ulusoy E., Aliyazıcıoğlu R. (2010). Comparative study of chemical and biochemical properties of different melon cultivars: Standard, hybrid, and grafted melons. J. Agric. Food Chem..

[39] Silveira A.C., Moreira G.C., Artés F., Aguayo E. (2015). Vanillin and cinnamic acid in aqueous solutions or in active modified packaging preserve the quality of fresh-cut Cantaloupe melon. Sci. Hortic..

[40] Calixto-Campos C., Carvalho T.T., Hohmann M.S. (2015). Vanillic acid inhibits inflammatory pain by inhibiting neutrophil recruitment, oxidative stress, cytokine production, and NFκB activation in mice. J. Nat. Prod..

[41] Rodríguez-Pérez C., Quirantes-Piné R., Fernández-Gutiérrez A., Segura-Carretero A. (2013). Comparative characterization of phenolic and other polar compounds in Spanish melon cultivars by using high-performance liquid chromatography coupled to electrospray ionization quadrupole-time of flight mass spectrometry. Food Res. Int..

[42] Chalker-Scott L., Fuchigami L.H. (2018). The role of phenolic compounds in plant stress responses. Plant Abiotic Stress.

[43] Xiong D., Chen J., Yu T., Gao W., Ling X., Li Y., Huang J. (2015). SPAD-based leaf nitrogen estimation is impacted by environmental factors and crop leaf characteristics. Sci. Rep..

[44] Yuan Z., Cao Q., Zhang K., Ata-Ul-Karim S.T., Tian Y., Zhu Y., Liu X. (2016). Optimal leaf positions for SPAD meter measurement in rice. Front. Plant Sci..

[45] Ignat I., Volf I., Popa V.I. (2011). A critical review of methods for characterisation of polyphenolic compounds in fruits and vegetables. Food Chem..

